# The effect of solution plasma treatment on the microbial safety and quality characteristics of albumen

**DOI:** 10.1002/fsn3.3807

**Published:** 2023-11-06

**Authors:** Gökhan Akarca, Ayşin Kahraman Avci

**Affiliations:** ^1^ Food Engineering Department Afyon Kocatepe University Afyonkarahisar Turkey

**Keywords:** albumen, foam stability, foaming capacity, pasteurization, *Salmonella* Enteritidis

## Abstract

The aim of this study was to make eggs microbially safe and increase their durability without damaging the functional properties of the albumen and preserving the solubility of its proteins as much as possible by the solution plasma technique. The pH, Brix, density, and viscosity values of samples decreased during treatment (*p* < .05 except pH). Although the L* and a* values of both the albumen and egg foam decreased, the b*, hue angle, and chroma values of both increased during treatment. The L* and a* values of the albumen decreased by 7.01 and 1.89 units, and the values of the egg foam decreased by 10.93 and 1.03 units, respectively. At the end of the treatment, foaming capacity and foam stability were decreased by 25% and 21.42%, respectively. Foaming capacity values obtained as a result of this treatment were higher and foam stability values were lower compared to the values obtained in pasteurization of eggs by heat treatment. The count of the two pathogenic bacteria inoculated into the albumen decreased during the treatment (*p* < .05), the count of *Salmonella* Enteritidis decreased to 0, and the count of *Staphylococcus aureus* decreased by 1.09 log cfu/g at the end of the treatment. Compared to current heat treatments, solution plasma treatment caused significantly less adverse effects on albumen quality characteristics. In particular, the foaming properties of the albumen were much less affected by this method and remained at higher values compared to the values achieved by other methods. The treatment also produced a microbiologically safer product.

## Graphical abstract

In this study, albumen was made microbially safe using the solution plasma technique, and their durability was increased without harming the functional properties of albumen and by preserving the solubility of its proteins as much as possible.

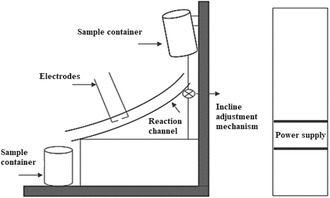

## 1 INTRODUCTION

Eggs are the only natural functional food containing all the nutritional factors necessary for the development of an embryo. The albumen, which constitutes about 58% of the egg, is mostly composed of water and has a total Brix value that generally varies between 11% and 13%. The second major component of eggs is protein. Eggs are a highly functional food due to its high protein content. Prior studies reported that albumen contains more than 40 protein types (Yuccer et al., 2012).

Albumen is widely used in the food industry in bakery products, desserts, and edible coatings in liquid or powder form for different purposes such as foaming, gel forming, binding (emulsifying), and volumizing. Under regular conditions, fresh, nonheat‐treated albumen can increase in volume sevenfold when beaten. Albumen is also the food item that has the strongest foaming capacity. The main protein contained in albumen that causes these properties is ovalbumin (Mine, 2002; Techer et al., 2015). Although it is possible to transform albumen into liquid form for industrial use by heat treatment, it denatures the proteins in them (Yuccer et al., 2012).

The desirable properties of albumen, such as foaming and emulsification, require high protein solubility. As the protein solubility of albumen decreases, so does its functionality. Protein solubility is affected by many factors such as staleness, heat treatment, and pH (Gomes & Pelegrine, 2012).

Treatment of albumen with thermal processes causes undesirable changes in the physicochemical and functional properties of the proteins. Since albumen is a viscous, protein‐rich, and highly hydrated medium, heat treatment causes deterioration in its components, resulting in nonenzymatic browning and coagulation of proteins (Campbell et al., 2003). Thermal denaturation causes polypeptide chains to break and allows these molecules to bond together to form aggregates or gel‐like structures. As heat treatment progresses, irregular clusters 0.1 μm in diameter form, increasing in number, and becoming denser with time (Akkouche et al., 2012).

Prior studies on this subject showed that thermal processing techniques increase the safety of eggs while decreasing their functionality. This is because a slight decrease in protein solubility significantly reduces the functionality of eggs (Gomes & Pelegrine, 2012). Heat treatment is still the most reliable technique for ensuring microbial safety in egg processing. However, while the use of this method ensures microbial reliability, it may cause negative effects on some quality performances of eggs. Although the effect of heat treatment on microorganisms varies according to the type of microorganism, it generally depends on the changes in the cell structure. Heat treatment microbial cell wall, cell membrane. It affects nucleic acids, structural enzymes, and proteins in the cell. As a result of heat treatment, cell damage occurs not with damage to a single target, but with the sum of damage to these components. This damage occurs in two ways, lethal and semi‐lethal. In cases where the heat treatment is lethal, the microorganisms are irreversibly eliminated. On the other hand, even if some of the microorganisms in the environment are damaged because of the heat treatments applied to the foods, they can survive and develop again with the repair mechanisms they have. This type of damage to microorganisms is called semi‐lethal damage. As a result of not storing processed foods in appropriate conditions, semi‐lethal‐damaged microorganisms may develop and cause problems. Bacteria such as *Salmonella* susp. *Escherichia coli*, and *S. aureus* may cause problems in this sense in the future, especially depending on the applied temperature and time relationship. The minimum heat treatment temperature and time for liquid albumen is 55.6°C for 6.2 min. At this minimum temperature, the solubility of albumen proteins decreases, especially affecting foaming and emulsifying properties and nutritional value negatively. Treating eggs at temperatures higher than this critical temperature and for longer than this critical time causes a film to form on the surface of the plates and coagulation. On the other hand, treating eggs at lower temperatures for less time makes the pasteurization process less effective (Unluturk et al., 2010).

Various pasteurization methods have been studied to extend the shelf life of liquid egg products, including ultrasonic wave treatment, high hydrostatic pressure, high electric field pulses, and ultra pasteurization and aseptic packaging. However, most of these are thermal methods and, like pasteurization, significantly deteriorates the functionality of liquid egg products (Atılgan & Unluturk, 2008).

Plasma is an ionized state of gas with a neutral charge distribution and charges moving in random directions. It is possible to convert matter from solid to liquid, from liquid to gas, and finally, from gas to plasma by transferring increasing amounts of energy to the relevant matter (Sakudo et al., 2019). Many studies on the use of plasma in disinfection and sterilization have shown that, in addition to reducing toxins, plasma can effectively neutralize microbial pathogens such as bacteria, fungi, and viruses. Different types of plasma such as high‐temperature plasma, thermal plasma, and nonthermal plasma can be used in various biological applications, including disinfection and sterilization. The solution plasma technique uses nonthermal plasma (Akyüz et al., 2016).

Solution plasma treatment is conducted in an open system and can be applied at a pressure of 1 atm without the need for an external gas supply. Although the characteristics of the power source used in the experiments vary according to a wide variety of factors such as solutions, electrode materials, plasma configurations, and volumes, the system elements consist of simple materials such as a power source, tungsten electrodes attached to ceramic or Teflon holders, and a reaction vessel (Özkan & Sakarya, 2019; Saito et al., 2018).

The power supply is used to adjust the pulse width, frequency, and voltage values during the application. Teflon holders are stable and cannot melt under plasma conditions, do not contaminate the solution with impurities, and ensure electrode retention. Tungsten is used in electrodes since it provides high stability, high chemical resistance, and high electric current. The cell in which all the reactions take place is called the reaction vessel (Akyüz et al., 2016).

The aim of the present study was to make eggs microbially safe and increase their durability without damaging the functional properties of the albumen, and preserving the solubility of its proteins as much as possible by using solution plasma.

## 2 MATERIALS AND METHODS

### 2.1 Materials

The eggs used were obtained from a producer in Afyonkarahisar Province, Türkiye (Afyon Yumurta/Turkiye). They were laid by *Gallus domesticus* hens, were white, and had the characteristics (permissible hidden cracks, class B, medium size) required by the standards used in pasteurized egg production.

### 2.2 Preparation of eggs for analysis

**FIGURE 1:**
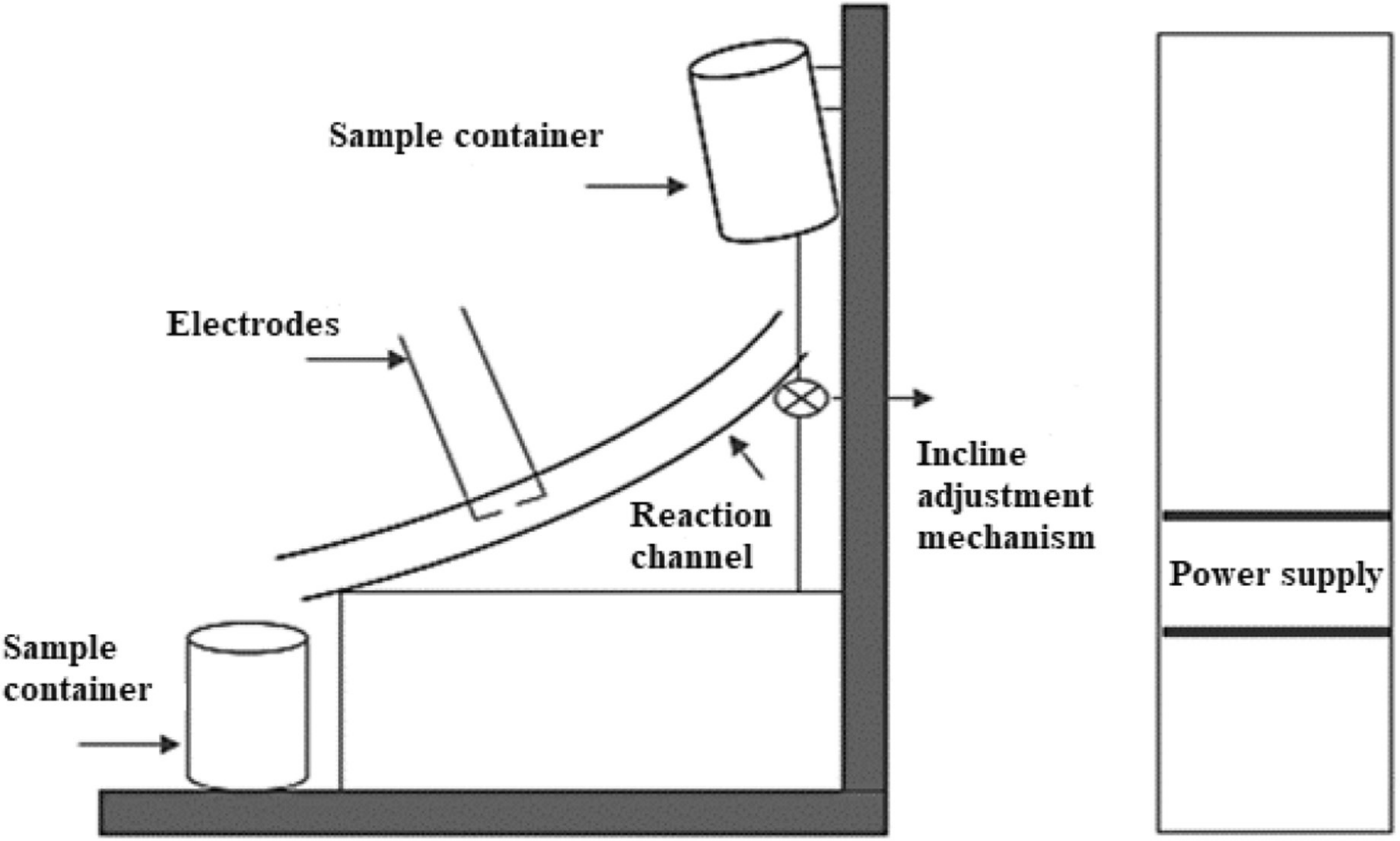
The system created for the application of the solution plasma technique in egg white.

The eggs were washed with sterile distilled water at 15°C before being broken open. After that, they were broken appropriately by hand and the yolk and albumen were separated without allowing them to mix. The albumen obtained was mixed in a homogenizer (Wisetis HG‐15D) at 16424 g for 1 min. The homogenized albumen was then placed into the prepared apparatus (Figure 1) and was treated with solution plasma. Flow rates and temperature data during the process applied to the samples are shown in Table 1.

**TABLE 1:** Flow rate and temperature data of samples.

NR	Amount (mL)	Starting temperature (°C)	Finish temperature (°C)	Transition time (s)	Amount per second (mL)
R	2000	17.2	19.0	58.28	0.029
T2	2000	19.0	20.7	59.11	0.030
T3	1750	20.7	21.4	50.13	0.029
T4	1450	21.4	22.5	42.25	0.029
T5	1200	22.5	22.7	33.32	0.028
T6	950	23.8	23.8	25.54	0.027
T7	700	23.8	24.3	18.47	0.026
T8	400	24.3	24.7	10.25	0.026

Abbreviation: NR, number of repetitions.

In this system, the distance between electrodes was measured using digital calipers (Mitutoyo, Ip‐67 0‐150, Japan) and set to 5 mm. The flow rate was set to 36.17 ± 2 mL/s, pulse width was set to 2.5 μs, voltage was set to 5 kV, frequency was set to 17,000 kHz, and slope was set to 66.70%. These parameters were kept constant throughout the experiment. During the experiment, albumen preparations were treated by the system eight times and samples were taken starting from the second treatment. For each repetition, the starting and ending temperatures (°C) and the amount of albumen (mL) passing through the reaction channel in 1 s were recorded.

### 2.3 Physicochemical analysis

#### 2.3.1 pH values of the samples

Ten grams of each sample was weighed with a precision balance (Sartorius CP224 S), 100 mL of pure water was added to the samples, and they were homogenized in a homogenizer (Wisetis HG‐15D) at 1800 rpm for 1 min. Then, after the samples reached room temperature (20 ± 2°C), pH values were measured and recorded with a pH meter (Hanna HI 2215) (Ayyash & Shah, 2011).

#### 2.3.2 Brix (%) values of the samples

To analyze the Brix (%) values of the albumen samples, they were first left until they reached room temperature (20 ± 2°C). The samples were then measured with a handheld refractometer (Atago 340‐32 B‐ATC, Japan). The value shown on the refractometer scale was recorded as the Brix (%) value of the sample (Yuceer, 2018).

#### 2.3.3 Density values of the samples

The densities of the albumen samples were measured using a densimeter. After the temperature‐adjusted samples were transferred to a graduated cylinder, the densimeter was slowly dropped into the sample without the neck of the device becoming wet. After the vertical oscillation of the densimeter stopped, the value on the densimeter was read by bringing the liquid surface to eye level (Anonymous, 2016). The density values of the samples were determined using the following equation:

$$d\left(g/cm^{3}\right)=\frac{1000+\text{value read in densimeter}}{1000}$$

#### 2.3.4 The viscosity values of the samples (mPa.s)

In the current study, the change in the viscosity of the albumen samples was analyzed using a viscometer (Brookfield, Middleboro, MA, USA) at 20 ± 2°C and 100 rpm using spindle number 2. Values determined at 15, 30, and 45 s were recorded (Shahnawaz & Shiekh, 2011). Viscosity was calculated using the equation presented below and was then converted to mPa.s, which is the main viscosity unit used in the literature, by making the necessary unit conversions (Erzengin, 2014).

$$\text{Viscosity}\;\left(\mathrm{Pa}\right)\;\left(\frac{\mathrm{kg}}{m.s}\right)=d\;\left(\frac{\mathrm{kg}}{m^{3}}\right)\times Q\;\left(\frac{m^{3}}{s}\right)\times \frac{1}{m}$$

#### 2.3.5 The color (L*, a*, b*, hue angle, and chroma) values of the samples

The color of the samples was measured using a colorimeter (Chroma Meter, CR‐400, Japan). The measured color values are L* (brightness, 0–100, black to white), a* (red‐green, −a* green, +a* red), and b* (yellow‐blue, −b* blue, +b* yellow) (35). Hue angle, which is used to measure color intensity, expresses the hue value of the color and chroma expresses the saturation value of the color (Gunısık, 2022).

The following equation was used to measure the hue angle (Mclellan et al., 1994):

Hueangle=180π×tan−1ba

The following equation was used to determine the chroma value:

Chroma value=a2+b212

#### 2.3.6 Foaming capacity, foam stability, and drainage volume values of samples

First, 100 mL of albumen samples adjusted to room temperature (20 ± 2°C) was mixed in a mixer (Mr Chef Quadro, Fakir) at 300 rpm/min for 2 min. The resulting foam was transferred with a sterile spatula to a 1000 mL graduated cylinder and the measured value was recorded as the foam volume (Yuceer, 2018). The following formula was used to calculate the foaming capacity (%) value.

$$\text{Foaming capacity}\;\left(\%\right)=100\times \frac{\text{foam volume}\;\left(\mathrm{mL}\right)}{\text{initial liquid phase volume}\;\left(\mathrm{mL}\right)}$$

Initial liquid phase volume: volume of albumen beaten in the mixer for analysis (mL).

In order to calculate foam stability (%), the foam was kept in the graduated cylinder for 1 h and the liquid accumulated in the graduated cylinder (drainage volume) was placed into a different 100 mL graduated cylinder and the accumulated amount was measured. The following equation was used to determine foam stability (%) (Yuceer, 2018):

$$\begin{array}{c} \text{Foam stability}\;\left(\%\right)=\\ 100\times \frac{\text{initial liquid phase volume}\;\left(\mathrm{mL}\right)-\text{drainage volume}\;\left(\mathrm{mL}\right)}{\text{initial liquid phase volume}\;\left(\mathrm{mL}\right)} \end{array}$$

### 2.4 Microbiological analyses

#### 2.4.1 Preparation of pathogenic bacteria and inoculation into the samples

*Staphylococcus aureus* (ATCC 6538) and *Salmonella* Enteritidis (ATCC 13076) strains provided by the American Type Culture Collection (ATCC, Rockville, MD, USA) were used. Single colonies of the test bacteria cultured on blood agar (Merck 110886, Germany) were inoculated into peptone water (Merck 115525) using a sterile loop and adjusted to 0.5 McFarland standard using a densitometer (8.17 Log cfu/mL) (Biosan 1B, Turkey). One milliliter of the prepared inoculum was inoculated into albumen samples.

#### 2.4.2 Count of *Salmonella* Enteritidis

Albumin samples inoculated with *Salmonella* Enteritidis and treated with solution plasma were placed in sterile Stomacher bags (Stomacher Lab Blender 400, London, UK). Then, 10 g of the samples in the bags were weighed on a precision balance (Laboratory Balances, Radwag PS R2.H, Poland) under sterile conditions and placed in another sterile Stomacher bag. Next, 90 mL of buffered peptone water (BPW) (Oxoid, CM 0509) solution was added to the samples and the mixture was homogenized in a Stomacher blender (BagMixer® 400 P‐080921247) for 2 min. The mixture was then incubated at 37°C in aerobic conditions for 24 h. Following incubation, 1 mL of the contents of the bags was extracted with a sterile automatic pipette (Eppendorf Research Plus) and transferred into tubes containing 9 mL of Rappaport Vassiliadis *Salmonella* enrichment broth (RVS) (Merck, 107666) and 9 mL of sterile Muller–Kauffmann tetrathionate/novobiocin broth (MKTTn) (Oxoid, CM 1048). Thus, a 10^−2^ dilution was prepared. Similarly, 1 mL of mixture was removed from these tubes and transferred to tubes containing 9 mL of RVS and MKTTn and 10^−3^ dilutions were prepared. Serial dilutions were prepared via similar procedures.

The tubes were then incubated at 43°C and 37°C for 24 h. After incubation, 0.1 mL of dilute solutions were taken from both tubes and transferred to xylose lysine deoxycholate agar (XLD) (Oxoid, CM 469) and brilliant green agar (BGA) (Oxoid, CM 0329) using a sterile tipped pipette. After being spread homogeneously with a sterile Drigalski spatula (Orlab, Turkey), both agars were incubated in an incubator (Incucell, MMM, Germany) at 37°C for 24 h. After incubation, black‐centered and pink colonies on XLD agar and pink and transparent colonies on BGA were considered suspicious (ISO, 2002).

At least three of these colonies of each sample were obtained with the help of a sterile loop and transferred to nutrient agar (Oxoid, CM 0003) and incubated at 37°C for 24 h. For biochemical tests, colonies growing on nutrient agar were transferred to triple sugar iron agar (TSIA) (Oxoid, CM 277) and lysine iron agar (LIA) (Oxoid, CM 381) using a sterile loop and incubated at 37°C for 24 h. Tubes showing color change were considered positive and these colonies were subjected to biochemical tests (Flowers et al., 1992).

In the biochemical evaluations, IMViC (indole, methyl red, Voges–Proskauer, citrate) test; H_2_S production; urease test; citrate utilization; ability to ferment glucose, lactose, maltose, arabinose, sorbitol, mannitol, and dulcitol; and Gram staining tests were performed on all suspicious colonies. Suspicious colonies were also tested for agglutination using *Salmonella* antiserum (Salmonella O Poly A‐1 and Vi‐Difco 2264‐47‐2) and colonies showing agglutination were considered positive (ISO, 2002).

#### 2.4.3 Count of *Staphylococcus aureus*

First, 10^−2^ dilutions of samples were prepared by placing 1 mL of the samples in Stomacher bags using a sterile pipette and inoculating them into tubes containing 9 mL of buffered peptone water (Merck Millipore, 107228, Germany). Serial dilutions were prepared via similar procedures and these dilutions were analyzed.

*Staphylococcus aureus* was counted using a mixture of Baird–Parker agar (Merck, 1.05406) and egg yolk tellurite emulsion (Merck, 103785). The inoculated Petri dishes were incubated under aerobic conditions at 30 and 35°C for 24 and 48 h, respectively. At the end of the incubation period, samples were taken from the suspicious colonies (black colonies with a white zone around them) with the help of a sterile loop and inoculated again on a mixture of Baird–Parker agar and egg yolk tellurite emulsion and left to incubate for the same period under the same conditions. Samples were taken from the incubated colonies and a coagulase test was performed with Bactident coagulase (Merck 1.13306) and positive results were considered *Staphylococcus aureus* (Bennett & Lancette, 2001).

### 2.5 Statistical analyses

The results obtained in the research were analyzed twice using two samples for each analysis. SPSS V 23.0.0 (SPSS Inc., Chicago, IL, USA) was used. The data obtained as a result of the analyses were evaluated by analysis of variance. The accepted significance level was determined by the Duncan test (*p* < .05). In addition, the effects of the results obtained were analyzed by Pearson correlation coefficient.

## 3 RESULTS AND DISCUSSION

### 3.1 Physicochemical analyses

#### 3.1.1 Changes in the pH, Brix, density, and viscosity of the samples

The pH, Brix, density, and viscosity values of the albumen samples decreased during the solution plasma treatment (*p* < .05). Although pH values did not decrease significantly during the first five repetitions, they decreased rapidly during the subsequent ones and were 8.6 ± 0.03 at the end of the last repetition.

**FIGURE 2:**
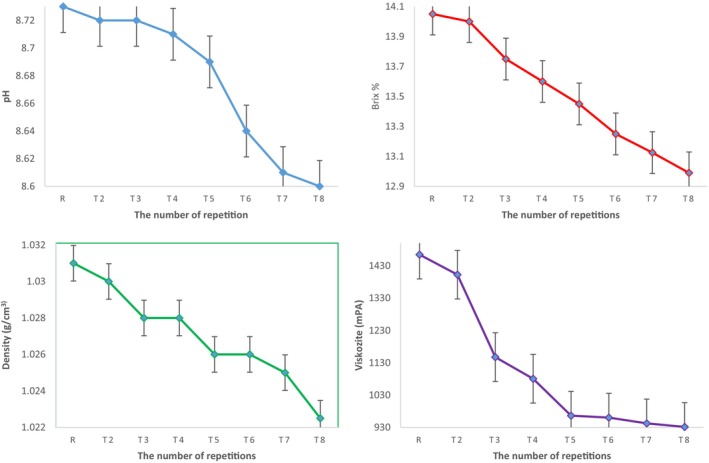
Changes in physicochemical values of egg samples during solution plasma processing.

The increase in H^+^ ions during the treatment affected the decrease in pH. Reactive substances with mainly acidic properties such as nitric acid (HNO_3_) and nitrous acid (HNO_2_) produced by plasma are responsible for the pH decrease during treatment (Akarca et al., 2023). Similarly, Akarca et al. (2022) reported that solution plasma treatment of raw milk decreased its pH value. The Brix value of the mixture, which was 14.05% before the solution plasma application, decreased to 12.99% after eight repetitions (Figure 2). Similarly, pretreatment density values of 1.031 g/cm^3^ and viscosity values of 1464.5 mPA decreased to 1.022 g/cm^3^ and 931.5 mPA after eight repetitions.

During the plasma treatment of the solution, the amount of solid albumen decreased due to transformation into liquid albumen, n‐ovalbumin transformed into s‐ovalbumin, which is less hydrophobic, and ovomucin–lysozyme bonds were broken. These factors affected the decrease in the Brix, density, and viscosity values of the samples (Ragni et al., 2007; Yuceer, 2018).

In their study in which they applied irradiation to liquid albumen, Song et al. (2009) found that the viscosity of the albumen decreased after the treatment, and they stated that this occurred due to protein breakdown. The decrease in the number of peptide bonds due to chain cleavage also decreases viscosity (Ho et al., 2021). Examination of the findings of the current study revealed that there was a sudden decrease in viscosity especially after the second repetition (Figure 2). Similar to prior studies in the literature, in the present study, the proteins in albumen were degraded due to environmental factors.

#### 3.1.2 Changes in color values of the samples

Sample type interaction; affected too much significantly the change of albumin and foam color values (except L*) (*p* < 0.0001). In addition, sample type showed negatively corelative effect on a* value, while it showed positively too much corelative effect on b*, hue angle, and chroma values (*p* < 0.01) (Tables 2 and 3).

**TABLE 2:** Color (L*, a*, and b*) values of albumin of egg samples during the solution plasma processing.

NR	L* value	a* value	b* value	Hue angle	Croma
R	66.39 ± 3.45^a^	5.96 ± 0.17^a^	7.17 ± 0.09^e^	50.27 ± 0.30^g^	9.32 ± 0.21^e^
T2	64.29 ± 3.07^a^	5.74 ± 0.14^ab^	9.90 ± 0.85^d^	59.78 ± 2.41^f^	11.45 ± 1.11^d^
T3	64.05 ± 0.87^a^	5.71 ± 0.06^b^	11.33 ± 0.02^c^	63.25 ± 0.17^e^	12.69 ± 0.05^c^
T4	63.94 ± 3.66^a^	5.38 ± 0.08^c^	12.97 ± 0.04^b^	67.47 ± 0.23^d^	14.04 ± 0.08^b^
T5	62.35 ± 2.44^a^	5.23 ± 0.11^cd^	13.67 ± 0.05^b^	69.06 ± 0.32^cd^	14.64 ± 0.10^b^
T6	61.59 ± 1.06^a^	5.13 ± .0.03^de^	13.86 ± 0.32^b^	69.68 ± 0.51^c^	14.78 ± 0.44^b^
T7	59.84 ± 8.65^a^	4.99 ± 0.04^e^	15.78 ± 0.03^a^	72.45 ± 0.18^b^	16.55 ± 0.03^a^
T8	59.38 ± 2.70^a^	4.07 ± 0.07^f^	16.30 ± 0.07^a^	75.98 ± 0.31^a^	16.80 ± 0.08^a^
*p* Value	.897	<.0001	<.0001	<.0001	<.0001
*r*	.483	−.911**	.966**	.947**	.966**

Abbreviations: NR, number of repetitions, ns, not statistically significant, *r*, correlation coefficient.

*Note*: a–f(↓), values with the same superscript letters in the same column for each analysis differ significantly (*p* < .05). *p* < .0001: Statistically too much significant, *p* < .01: Statistically too significant, *p* < .05: Statistically significant, *p* >.05: Not statistically significant, *p < .05; **p < .01.

**TABLE 3:** Color values of foam of egg samples during the solution plasma processing.

NR	L* value	a* value	b* value	Hue angle	Croma
R	92.37 ± 2.11^a^	3.95 ± 0.10^a^	−2.49 ± 0.04^e^	327.77 ± 1.09^d^	4.67 ± 0.06^a^
T2	90.17 ± 2.80^a^	3.86 ± 0.03^a^	−2.44 ± 0.08^e^	327.70 ± 1.08^d^	4.57 ± 0.02^a^
T3	88.07 ± 5.48^ab^	3.85 ± 0.08^a^	−2.16 ± 0.01^d^	330.70 ± 0.38^cd^	4.42 ± 0.08^b^
T4	87.62 ± 3.35^ab^	3.65 ± 0.01^b^	−2.12 ± 0.08^d^	329.86 ± 1.09^cd^	4.22 ± 0.03^c^
T5	87.15 ± 5.21^ab^	3.57 ± 0.06^b^	−2.10 ± 0.03^d^	329.53 ± 0.74^d^	4.15 ± 0.04^c^
T6	86.10 ± 1.26^ab^	3.53 ± 0.04^b^	−1.70 ± 0.03^c^	334.29 ± 0.64^bc^	3.92 ± 0.03^d^
T7	81.54 ± 1.56^b^	3.38 ± 0.01^c^	−1.48 ± 0.07^b^	336.36 ± 1.10^b^	3.69 ± 0.01^e^
T8	81.44 ± 0.52^b^	2.92 ± 0.07^d^	0.44 ± 0.07^a^	373.09 ± 4.80^a^	2.96 ± 0.06^f^
*p* Value	.091	<.0001	<.0001	<.0001	<.0001
*r*	−.810**	−.928**	.819**	.707**	−.919**

Abbreviations: NR, number of repetitions; ns, not statistically significant; *r*, correlation coefficient.

*Note*: a–e(↓): Values with the same superscript letters in the same column for each analysis differ significantly (*p* < .05). *p* < .0001: Statistically too much significant, *p* < .01: Statistically too significant, *p* < .05: Statistically significant, *p* > .05: Not statistically significant, *p < .05; **p < .01.

During the solution plasma treatment, the L* and a* values of the albumin and foam of the egg samples decreased, whereas the b* values increased. The hue angle and chroma values also increased in parallel with these values (*p* < .05).

The acidic substances formed during solution plasma treatment and the CO_2_ in the composition of albumen cause turbidity, which leads to a decrease in L* values. Studies in the literature reported that the force of the electrostatic repulsion field in albumen is responsible for its clarity. The fact that the initial pretreatment L* value was significantly higher after the solution plasma treatment indicates that the clarity of the albumen was high, which means that initially the proteins could have been uniformly dispersed in the bulk phase under electrostatic repulsion and no aggregation occurred (Haobo et al., 2023; Ji'en et al., 2022).

During solution plasma treatment, denaturation of some proteins, changes in electrical charges, and changes in electrostatic balance resulted in a gradual decrease in L* values. In their study on the foam properties of multiple proteins, Ji'en et al. (2022) reported that uniform protein distribution promotes the migration and stabilization of protein molecules toward the air–water boundary. They stated that protein ratios also affected turbidity and that the presence of ovomucoid especially affected the protein distribution in the bulk solution (Haobo et al., 2023). Increasing numbers of solution plasma treatment repetitions caused the interfacial adsorption behavior of multiple proteins to change, disrupting the uniform distribution of proteins, and decreasing the foaming capacity of the samples as well as their L* values. In the current study, the albumen foam samples had higher L* values than the liquid albumen samples. This result is similar to those reported in prior studies (Gunısık, 2022).

The a* values of the liquid albumen and albumen foam samples gradually decreased with further treatment. As the number of repetitions increased, browning of the samples increased as a result of the increasing temperature. The decrease in the b* values of the egg samples indicated yellowing due to the increasing heat and was consistent with the decrease in the a* value. The changes observed in the a* and b* values of the samples are like the results of prior studies (Gunısık, 2022).

The fact that the increase in chroma values of the liquid albumen samples processed by solution plasma is similarly positively correlated with the number of treatments and temperature increase indicates that the samples were turning from white to yellow. The lower chroma values of the albumen foam compared to the liquid albumen observed in the present study are like the results reported by Gunısık (2022).

#### 3.1.3 Changes in the foaming capacity and foam stability of the samples

Foaming capacity and foam stability are among the most important indicators of the functionality of albumen (Yuceer, 2018). The sample type affected the foaming capacity very significantly (*p* < .0001). Sample type had a negative correlation with foaming capacity and foam stability and a positive correlation with drainage volume (*p* < .01) (Table 4).

**TABLE 4:** Physical properties of egg foam samples during the solution plasma processing.

NR	Foam capacity (%)	Foam stability (%)	Drainage volume (mL)
R	800 ± 14.13^a^	70 ± 5.65^a^	30 ± 5.65^d^
T2	750 ± 42.42^ab^	69 ± 2.82^ab^	31 ± 2.82^cd^
T3	750 ± 14.14^ab^	63 ± 7.07^abc^	37 ± 7.07^bcd^
T4	700 ± 0.00^b^	54 ± 1.41^cd^	46 ± 1.41^ab^
T5	700 ± 14.14^b^	59 ± 4.24^bcd^	41 ± 4.24^abc^
T6	610 ± 28.28^c^	50 ± 2.82^d^	50 ± 2.82^a^
T7	600 ± 0.00^c^	52 ± 2.82^cd^	48 ± 2.83^ab^
T8	600 ± 28.28^c^	55 ± 5.65^cd^	45 ± 5.65^ab^
*p* Value	<.0001	.011	.011
*r*	−.944**	−.783**	.783**

Abbreviations: NR, number of repetitions; ns, not statistically significant; *r*, correlation coefficient.

*Note*: a–d(↓): Values with the same superscript letters in the same column for each analysis differ significantly (*p* < .05). *p* < .0001: Statistically too much significant, *p* < .01: Statistically too significant, *p* < .05: Statistically significant, *p* > .05: Not statistically significant, **p < .01.

Solution plasma treatment of the samples resulted in a decrease in foaming capacity and foam stability, but an increase in drainage volume (*p* < .05). Due to the treatment, foaming capacity and foam stability decreased by 25% and 21.42%, respectively. Drainage volume, which was 30 mL before the treatment, increased to 50 mL at the end of the sixth repetition and then decreased with the subsequent repetitions, reaching 45 mL at the end of the treatment (*p* < .05) (Table 4).

Many factors influence the characteristics and structure of albumen foam. They include albumen beating time and speed, temperature, homogenization, protein content and concentration, pH, storage time, and fat, acid, copper, salt, and sugar content (Henry & Barbour, 1933).

The ideal protein compositions for foam volume and stability require protein types with low molecular weight, high surface hydrophobicity, and good solubility (Belitz et al., 2004). It is reported that ovalbumin and ovomucoid are the key proteins in albumen protein that affect foaming capacity and foam stability. These proteins have good solubility and are highly electronegative. The composition of proteins in albumen affects foaming to a great degree, and it is known that environmental factors affecting the net load of proteins also directly affect foaming capacity. An example of this is the positively charged lysozyme interacting with negatively charged proteins at the interface and causing electrostatic change. In addition, the high viscosity of ovomucoid protein in albumen and the film formed by ovomucin between the liquid layer and air bubbles also have a protective effect on the stability of the foam (Haobo et al., 2023; Ji'en et al., 2022; Pernell et al., 2000; Sagis et al., 2001). Ovomucoid consists of three polymer structures containing nine disulfide bonds. Previous research has shown that ovomucoid has a high resistance to environmental factors. The findings of our study show that surface hydrophobicity increased due to the decrease in viscosity and density. Despite this, the foaming capacity decreased, and foam stability was maintained at 14.32% higher than foaming capacity. This suggests that the parameters applied in the solution plasma technique denatured other protein components without denaturing ovomucoid. In a study conducted by Haobo et al. (2023) in which key proteins affecting the foaming ability of albumen were examined, ovomucoid and lysozyme proteins highly affected the maintenance of foam stability. This finding also supports the findings of the current study. Thus, it can be concluded that the environmental factors applied to the samples undergoing solution plasma treatment did not have a negative effect on ovomucoid.

The solution plasma treatment applied to the albumen caused the electrical charges of the proteins in the egg, which have a positive effect on the foam, to change and denature. This resulted in a decrease in protein solubility, deterioration of the film structure, and a decrease in viscosity, resulting in a decrease in foaming capacity and foam stability. In addition, lower pH values due to treatment also decreased foaming capacity and foam stability. Beating time and speed caused an increase in foam volume, but a direct decrease in foam density and stability. Prolonged beating may result in excessive dissolution of the ovomucin and reduced elasticity of the air bubbles due to mechanical deformation (Van der Plancken et al., 2007).

#### 3.1.4 Relationship between the foaming capacity and physicochemical properties of albumen samples

**FIGURE 3:**
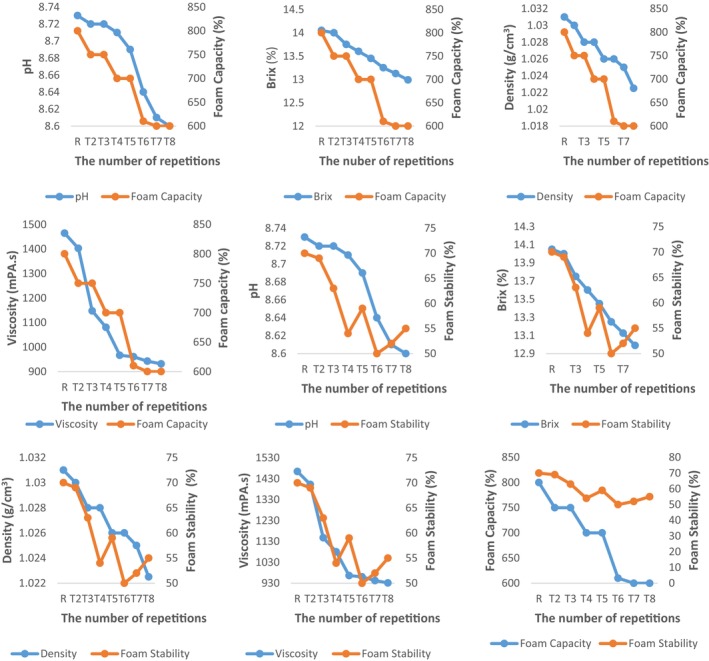
The relationship between foaming capacity and stability and physicochemical properties of albumen samples.

Foaming capacity and foam stability decreased in parallel with the decrease in pH, Brix, density, and viscosity of albumen samples as the number of solution plasma treatment repetitions increased (Figure 3). Foam capacity showed positively too much correlative effect on all physicochemical properties (*p* < .01). In addition, foam stability showed positively much correlative effect on density value (*p* < .05), while it showed positively too much correlative effect on Brix value (*p* < .01; Table 5). The re‐exposure of the albumen to the electrical field during each repetition of the process caused the electrical charges of the samples to change, denaturing the proteins, decreasing the solubility of proteins, and disrupting the film structure, which ultimately caused a decrease in the foam properties. As shown in many studies, the foam properties of albumen vary according to a wide range of factors. Foam properties mainly depend on protein molecular structure and competitive‐synergistic adsorption and cannot be directly correlated with environmental factors. These results prove once again that the macroscopic properties of ovomucoid/ovalbumin complex systems, especially found in mixed biomolecular systems such as eggs, are not determined by a specific factor but by multiple factors. While in some studies decreased viscosity in eggs increased foaming capacity by expanding the surface area, in others as viscosity decreased the gel structure deteriorated, the amount of solid albumen decreased, and the ratio of liquid albumen increased, ultimately decreasing foaming capacity (Haobo et al., 2023; Ji'en et al., 2022; Sarıbay & Köseoğlu, 2012b).

**TABLE 5:** Correlation analysis of foaming capacity and stability and physicochemical properties.

	Physicochemical properties		
	pH	Density	Brix
Foam capacity	.670**	.707**	.731**
Foam stability	.431	.605*	.754**

Abbreviation: *r*, correlation coefficient.

*Note*: *p < .05; **p < .01.

**FIGURE 4:**
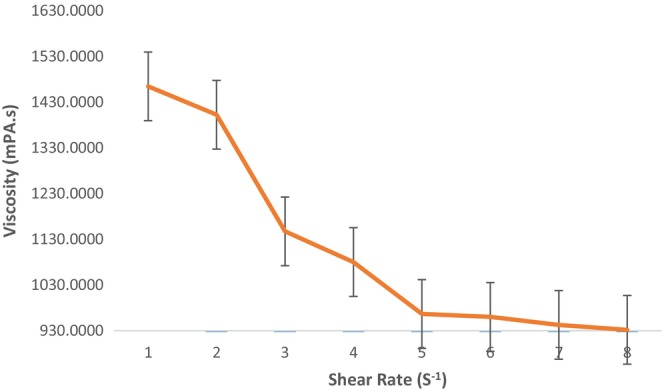
Relationship between shear rate and viscosity.

The albumen viscosity values produced a shear rate that caused pseudoplastic fluid behavior. This finding is like the results Atılgan and Unluturk (2008) found (Figure 4). Solution plasma treatment had no effect on the fluid behavior of albumen. As non‐Newtonian fluids' viscosity decreases, their shear rate increases.

### 3.2 Microbiological analysis results

In albumen samples inoculated with bacteria, bacterial counts decreased after the solution plasma treatment (*p* < 0.05). Pathogenic bacteria can have two different cell wall properties, Gr (−) and Gr (+). Gr (−) *Salmonella* Enteritidis growth in the albumen samples was not detected after the eighth repetition. On the other hand, although the count of Gr (+) *Staphylococcus aureus* decreased during treatment, 1.09 log cfu/g of this bacterium remained in the samples after the eighth repetition (Table 6). The difference between the two bacteria was due to their cell wall properties. The peptidoglycan layer and acid molecules present in the outer part of the cell wall of Gr (+) bacteria protected the cell from the effects of solution plasma treatment. In Gr (+) *Staphylococcus aureus*, 50%–90% of the cell wall is composed of a very thick layer of peptidoglycan and tetrapeptide chains that increase the strength of the peptidoglycan. The cell wall of the Gr (−) *Salmonella* Enteritidis contains roughly 10% peptidoglycan in its structure and the remaining part of the cell wall is a thin layer. For this reason, the destruction of Gr (−) bacteria in non‐thermal treatments such as solution plasma treatment and PEF treatment occurs faster than the destruction of Gr (+) bacteria (Koneman et al., 1992; Yalçın, 2018).

**TABLE 6:** Change in pathogen bacteria counts during the solution plasma processing (log cfu/g).

NR	*Salmonella* Enteritidis	*Staphylococcus aureus*
R	6.89 ± 0.02^a^	7.01 ± 0.01^a^
T2	4.18 ± 0.01^b^	4.26 ± 0.20^b^
T3	3.99 ± 0.01^c^	4.21 ± 0.20^b^
T4	3.58 ± 0.15^d^	3.28 ± 0.40^c^
T5	2.09 ± 0.10^e^	2.76 ± 0.50^d^
T6	1.55 ± 0.11^f^	2.03 ± 0.20^e^
T7	0.39 ± 0.12^g^	1.37 ± 0.57^f^
T8	0^h^	1.09 ± 0.45^f^

*Note*: a–g(↓): Values with the same superscript letters in the same column for each analysis differ significantly (*p* < .05).

Abbreviation: NR, number of repetitions.

In the current study, foaming capacity and foam stability decreased by 25% and 21.42%, respectively, after eight treatments (the number of treatments required to completely inhibit *Salmonella* Enteritidis). This decrease in foaming capacity and foam stability that occurred despite no heat treatment being applied can be attributed to the low pretreatment values. The subsequent decrease in these values with increasing number of repetitions may have been due to the negative effect of the electrical field generated during the process on protein charges. Protein molecules tend to lose their solubility due to loss of electrostatic repulsion (Arwa, 2013; Foegeding et al., 2006).

Baba et al. (2018) compared PEF‐treated and untreated liquid albumen and reported that no morphological differences, including protein aggregation, were found between the microimages of the samples. In another study, PEF and heat treatment achieved the same level of microorganism inactivation, while protein denaturation did not occur in PEF‐treated eggs (Baba et al., 2018; Sampedro et al., 2006).

When compared with sonication, which is an alternative treatment method, solution plasma treatment had fewer negative effects on the solubility of albumen proteins and can be applied at a much lower cost. The biggest disadvantage of phosphorylation, another alternative method, is the difficulty of removing residual chemicals. This is not a problem in solution plasma treatment. Methods based on various enzyme treatments, on the other hand, are costly and can take 24–48 h, making them significantly disadvantageous compared to solution plasma treatment.

In addition, in an experimental study in which various additives were added to albumen, NaOH was the best additive, but NaOH had significant negative effects on the pH values of samples (Chaiyasit et al., 2019; Lomakina & Milkova, 2006). In another study, the foaming capacity of albumen subjected to 3 kGy of irradiation increased by 21.4% compared to the control but caused a serious decrease of 132% in stability (Sarıbay & Köseoğlu, 2012a, 2012b). Darvishi et al. (2012) reported that convectional heating at 60°C for 6.5 min greatly increased the L* value of samples. Ohmic heating at 60 Hz–30 V/cm voltage gradient also increased the brightness of samples to 80.10 L*. High hydrostatic pressure treatment, which was tested as another alternative treatment, at pressures of 300–450 mPa caused protein denaturation.

## 4 CONCLUSION

In the present study, the solution plasma technique was used to render eggs microbially safe and increase their durability without damaging the functional properties of the albumen and preserving the solubility of albumen proteins as much as possible. No practices based on the sterilization of albumen while preserving their foaming ability have been clearly identified. The pH, Brix, density, and viscosity values of the albumen samples decreased during treatment. Although the L* and a* values of both albumen and egg foam decreased, the b*, hue angle, and chroma values of both increased during treatment. In addition, the foaming capacity, foam stability, and drainage volume values decreased during the eight treatments. The count of the two pathogenic bacteria inoculated into the albumen decreased during the treatment, with the count of *Salmonella* Enteritidis decreasing to zero and the count of *Staphylococcus aureus* decreasing by 1.09 log cfu/g by the end of the eighth treatment. Compared to mainstream heat treatments, solution plasma treatment caused significantly less adverse effects on albumen quality characteristics. In particular, the foaming properties of the albumen were much less affected by this method and remained at higher values compared to the values achieved by other methods. The treatment also produced a much more microbiologically safe product.

The best results in the study were obtained with the application of T8. The solution plasma technique can be used as an alternative to heat treatment alone in albumen sterilization, as well as in combination with vacuum treatment, preheating, ultrafiltration, or additive treatment. The solution plasma technique, which effectively sterilizes albumen, is advantageous compared to other treatment techniques in the literature since it maintains foam stability. However, using this technique in combination with other methods identified in the literature would be more appropriate and may improve the 25% decrease in foaming capacity caused by this technique. Pretreatment ultrafiltration/dialysis may be an important step in increasing the efficiency of the solution plasma technique by increasing the foaming performance obtained in the current research.

Many studies have been conducted on the preservation of the functional properties of liquid albumen while extending their shelf life by ensuring their microbial safety through thermal pasteurization. However, new effective methods are still needed in the liquid egg sector. Studies in the literature reporting that there is no difference between bakery products made from PEF‐treated eggs and raw eggs are promising for the use of solution plasma treatment in albumen. The solution plasma technique has been introduced into the literature as a new method. Studies are needed to evaluate the integration of this method with a synergistic process and to evaluate its systematic applicability in the industrial egg sector.

## AUTHOR CONTRIBUTIONS

**Gökhan Akarca:** Conceptualization (equal); formal analysis (equal); investigation (equal); methodology (equal); writing – original draft (lead); writing – review and editing (lead). **Ayşin Kahraman Avci:** Formal analysis (equal); methodology (equal); writing – original draft (supporting).

## FUNDING INFORMATION

The authors declare that no funds, grants, or other support were received during the preparation of this manuscript.

## CONFLICT OF INTEREST STATEMENT

The authors declared no potential conflicts of interest with respect to the research, authorship, and/or publication of this manuscript.

## ETHICS STATEMENT

This article does not contain any studies with human participants or on animals performed by any of the authors.

## CONSENT TO PARTICIPATE

The corresponding author and all the co‐authors participated in the preparation of this manuscript.

## INFORMED CONSENT

For this type of study, formal consent is not required.

## Data Availability

The original data with the respective analysis corresponding to the results shown in this work are available up to reasonable requirements.
